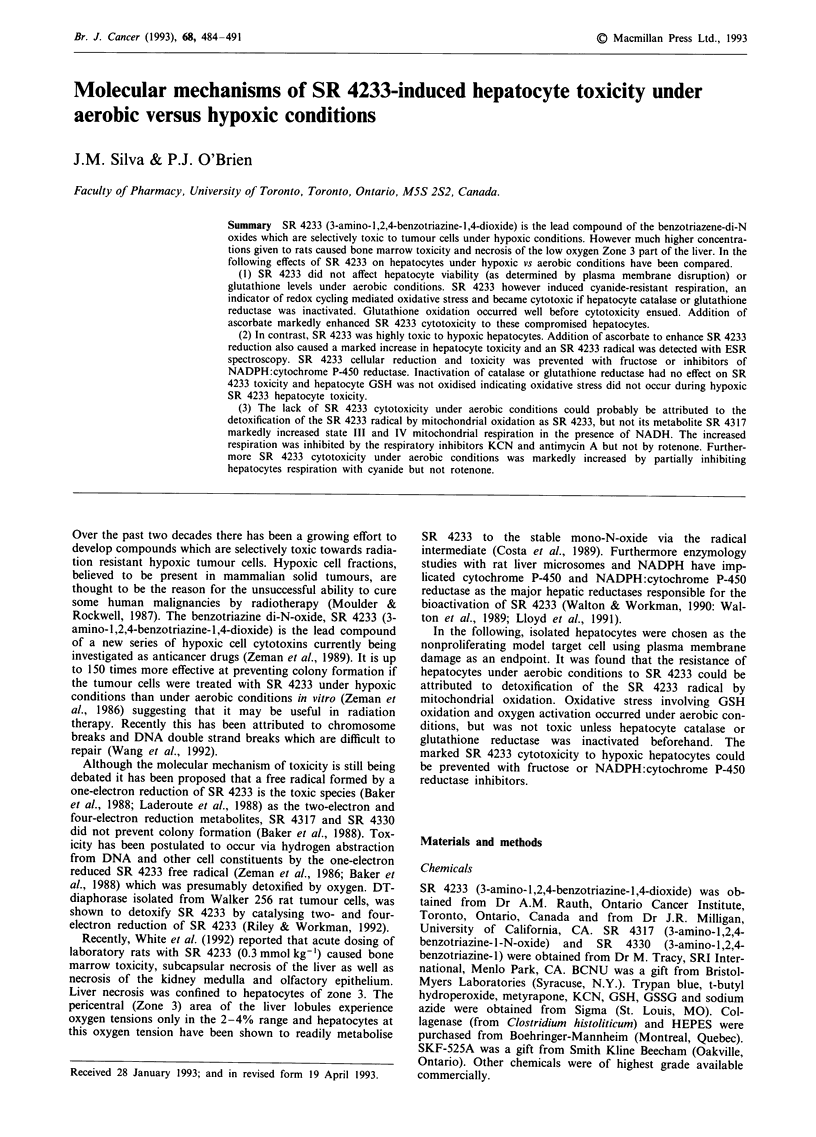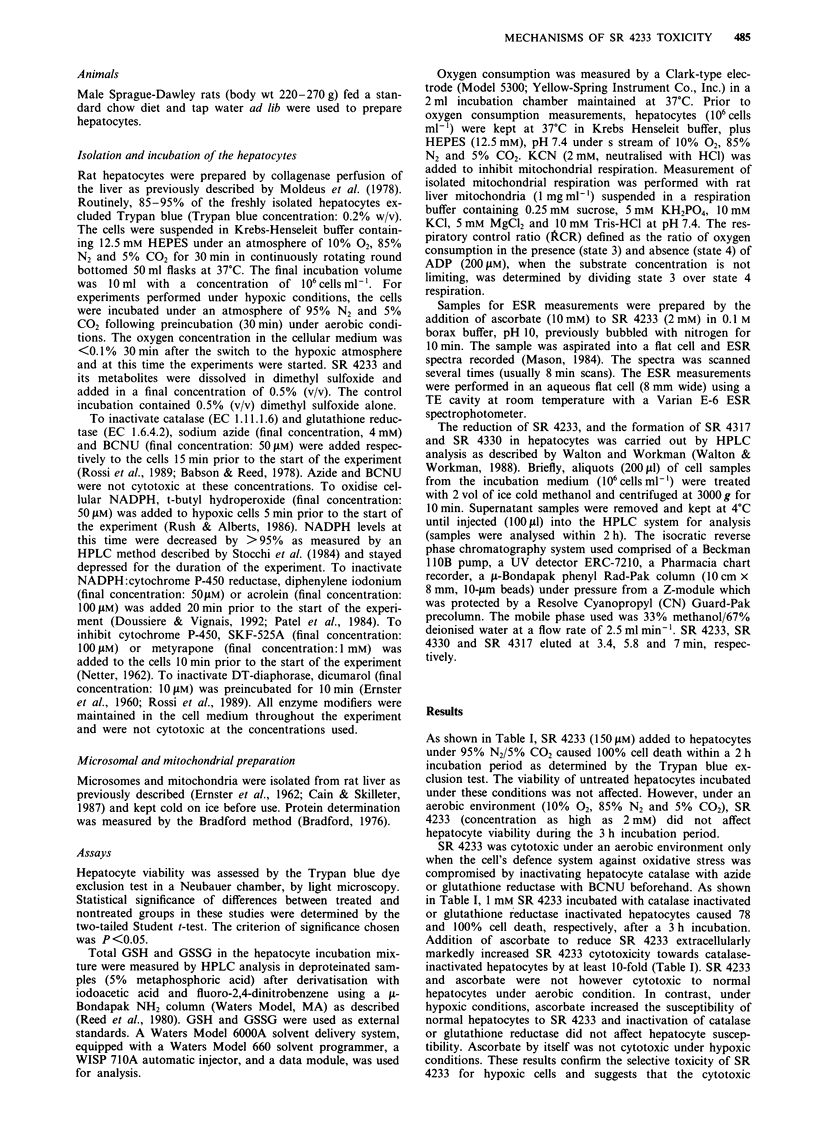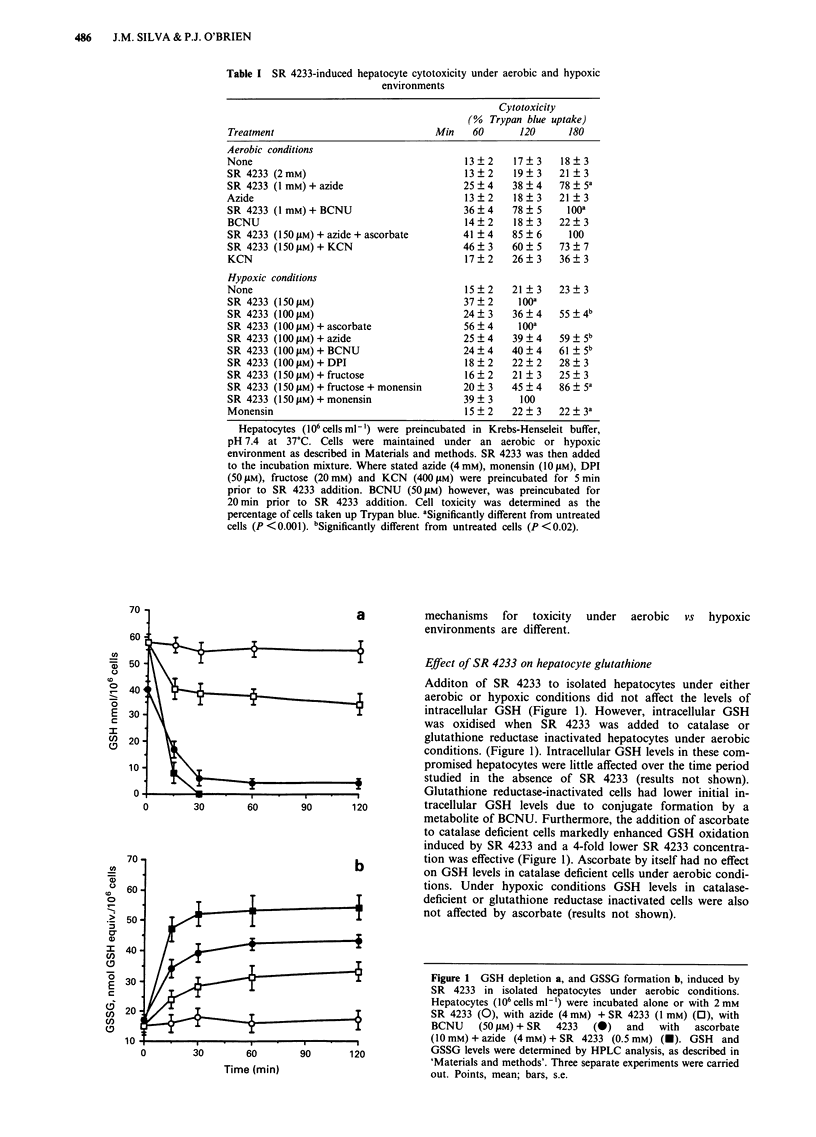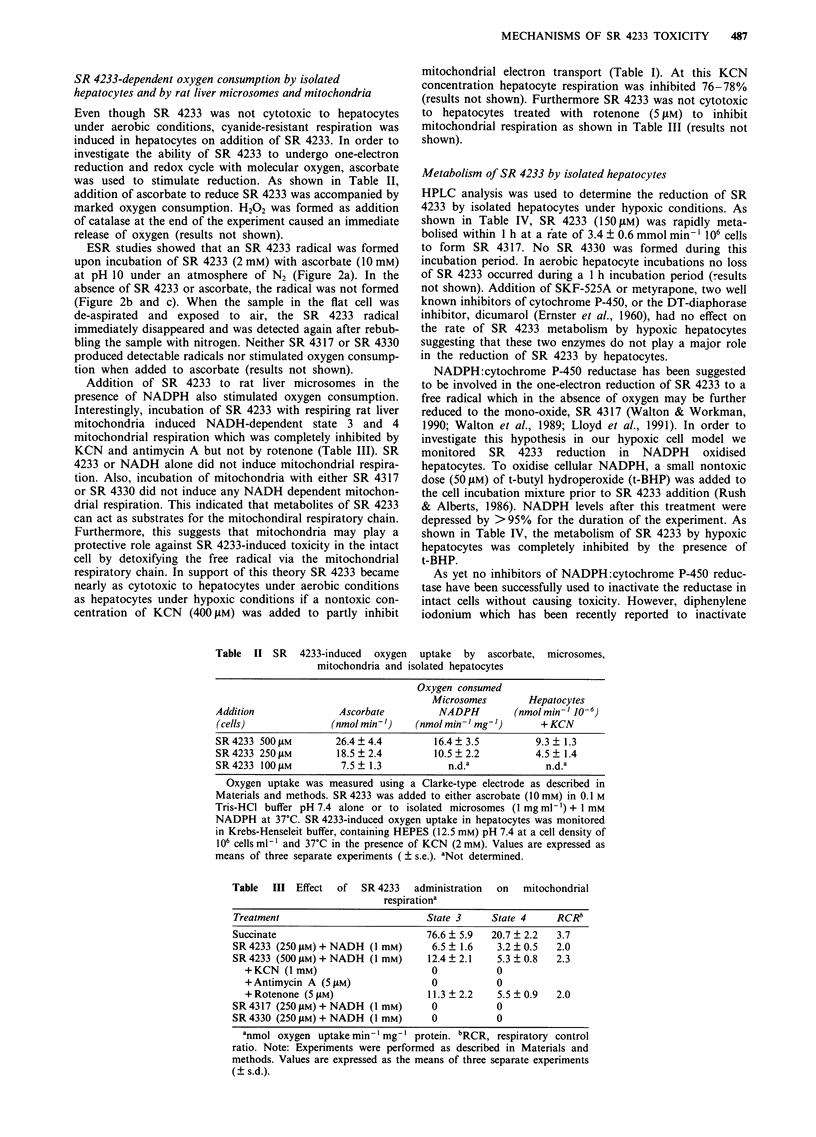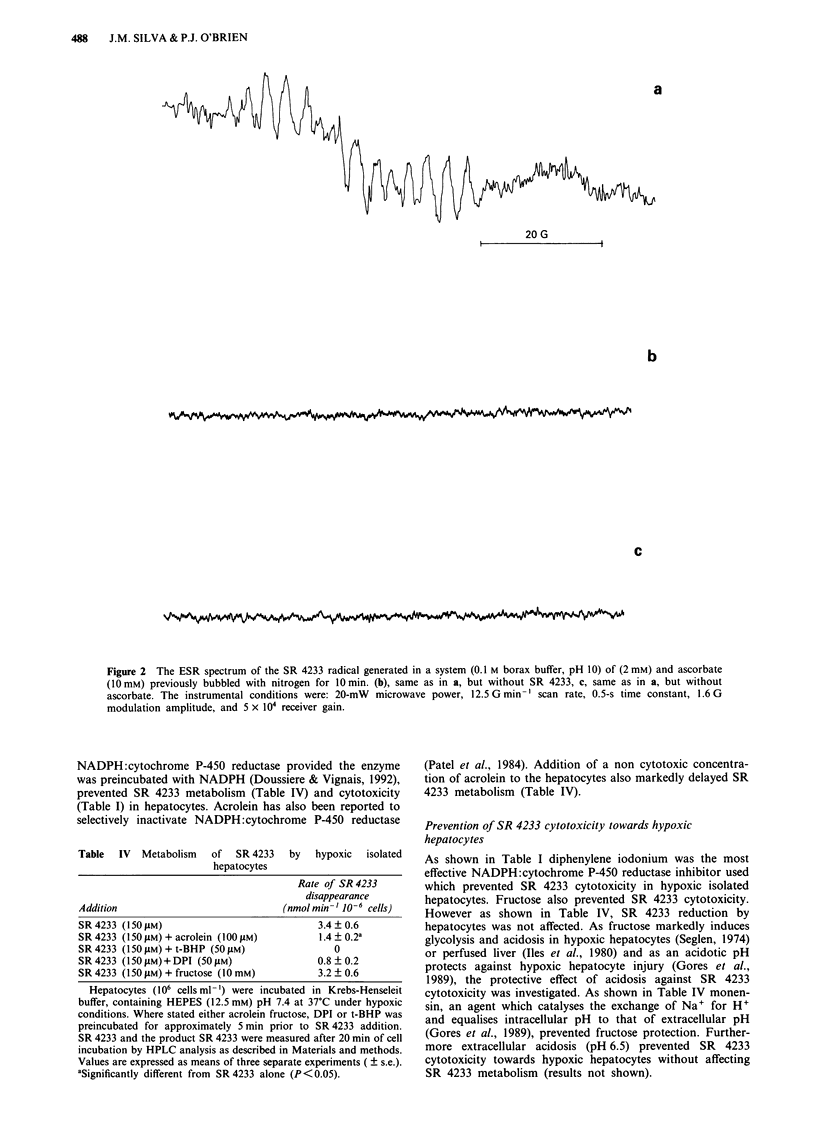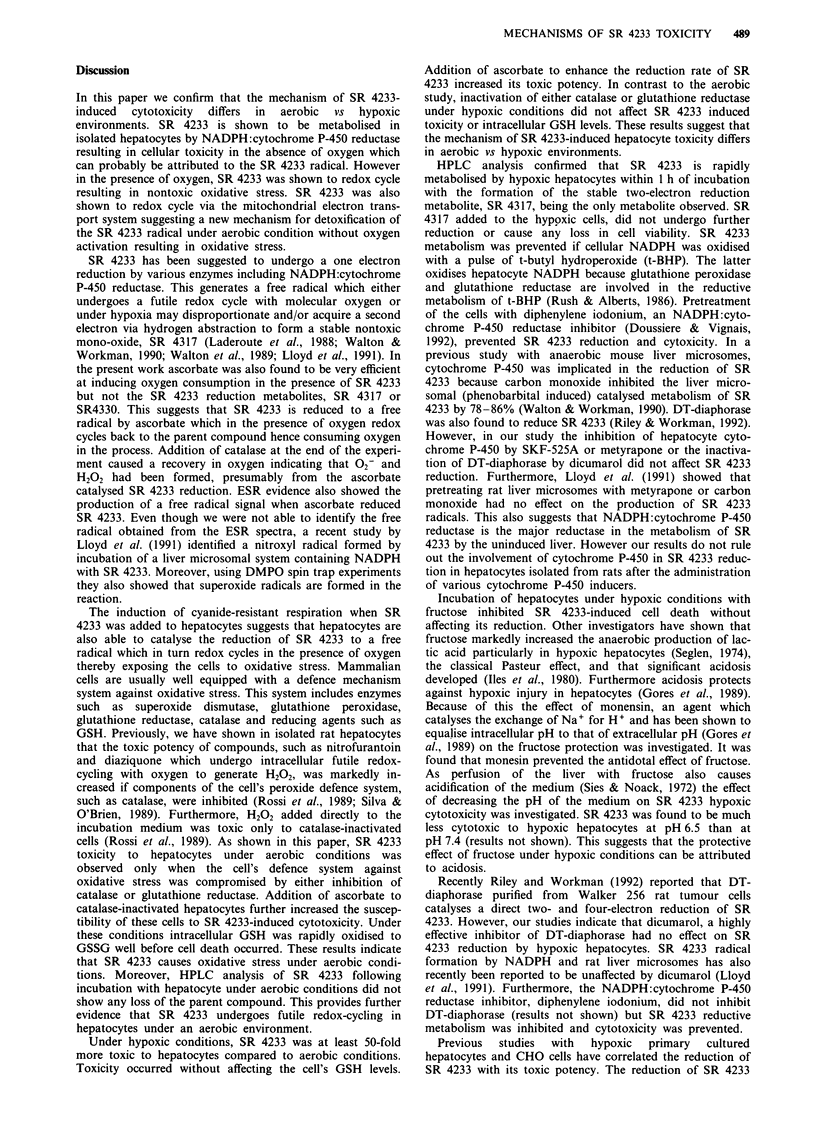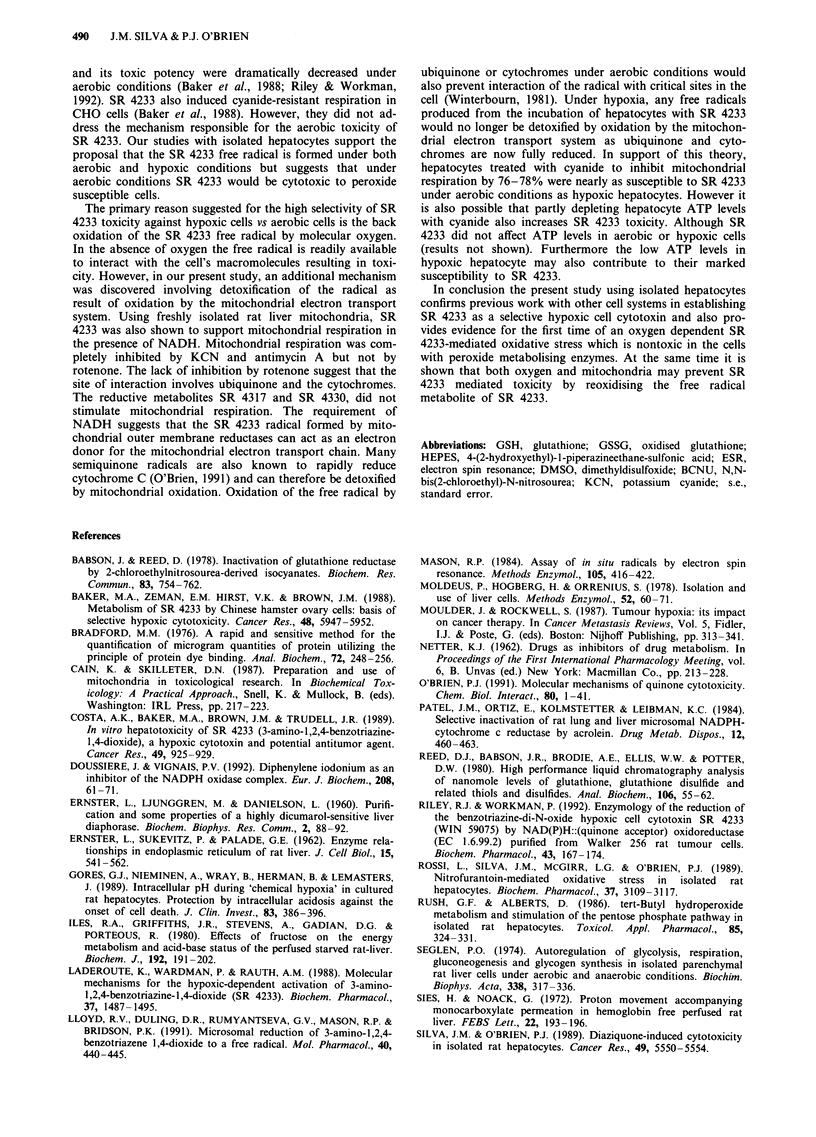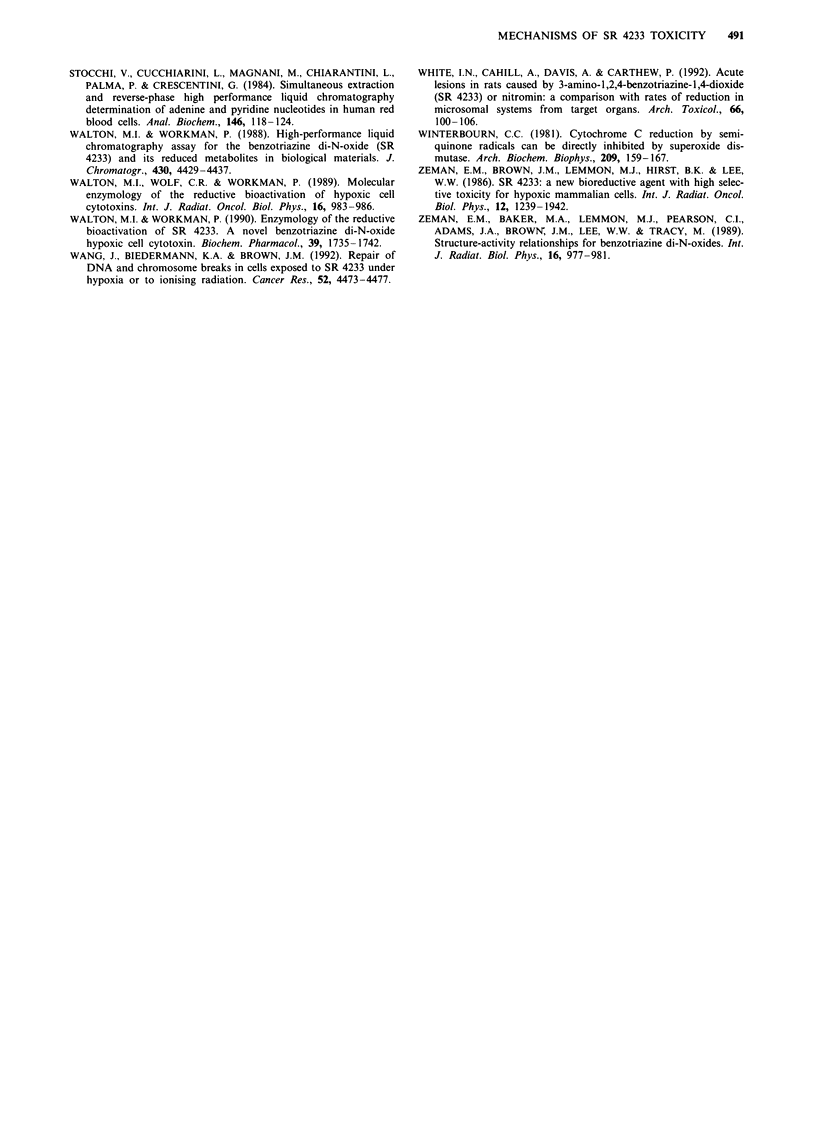# Molecular mechanisms of SR 4233-induced hepatocyte toxicity under aerobic versus hypoxic conditions.

**DOI:** 10.1038/bjc.1993.374

**Published:** 1993-09

**Authors:** J. M. Silva, P. J. O'Brien

**Affiliations:** Faculty of Pharmacy, University of Toronto, Ontario, Canada.

## Abstract

SR 4233 (3-amino-1,2,4-benzotriazine-1,4-dioxide) is the lead compound of the benzotriazene-di-N oxides which are selectively toxic to tumour cells under hypoxic conditions. However much higher concentrations given to rats caused bone marrow toxicity and necrosis of the low oxygen Zone 3 part of the liver. In the following effects of SR 4233 on hepatocytes under hypoxic vs aerobic conditions have been compared. (1) SR 4233 did not affect hepatocyte viability (as determined by plasma membrane disruption) or glutathione levels under aerobic conditions. SR 4233 however induced cyanide-resistant respiration, an indicator of redox cycling mediated oxidative stress and became cytotoxic if hepatocyte catalase or glutathione reductase was inactivated. Glutathione oxidation occurred well before cytotoxicity ensued. Addition of ascorbate markedly enhanced SR 4233 cytotoxicity to these compromised hepatocytes. (2) In contrast, SR 4233 was highly toxic to hypoxic hepatocytes. Addition of ascorbate to enhance SR 4233 reduction also caused a marked increase in hepatocyte toxicity and an SR 4233 radical was detected with ESR spectroscopy. SR 4233 cellular reduction and toxicity was prevented with fructose or inhibitors of NADPH:cytochrome P-450 reductase. Inactivation of catalase or glutathione reductase had no effect on SR 4233 toxicity and hepatocyte GSH was not oxidised indicating oxidative stress did not occur during hypoxic SR 4233 hepatocyte toxicity. (3) The lack of SR 4233 cytotoxicity under aerobic conditions could probably be attributed to the detoxification of the SR 4233 radical by mitochondrial oxidation as SR 4233, but not its metabolite SR 4317 markedly increased state III and IV mitochondrial respiration in the presence of NADH. The increased respiration was inhibited by the respiratory inhibitors KCN and antimycin A but not by rotenone. Furthermore SR 4233 cytotoxicity under aerobic conditions was markedly increased by partially inhibiting hepatocytes respiration with cyanide but not rotenone.


Br. J. Cancer (1993), 68, 484-491 © Macmillan Press Ltd., 1993

### Molecular mechanisms of SR 4233-induced hepatocyte toxicity under aerobic versus hypoxic conditions

J.M. Silva & P.J. O'Brien

*Faculty of Pharmacy, University of Toronto, Toronto, Ontario, M5S 2S2, Canada.*

**Summary** SR 4233 (3-amino-1,2,4-benzotriazine-1,4-dioxide) is the lead compound of the benzotriazene-di-N oxides which are selectively toxic to tumour cells under hypoxic conditions. However much higher concentrations given to rats caused bone marrow toxicity and necrosis of the low oxygen Zone 3 part of the liver. In the following effects of SR 4233 on hepatocytes under hypoxic *vs* aerobic conditions have been compared.

(1) SR 4233 did not affect hepatocyte viability (as determined by plasma membrane disruption) or glutathione levels under aerobic conditions. SR 4233 however induced cyanide-resistant respiration, an indicator of redox cycling mediated oxidative stress and became cytotoxic if hepatocyte catalase or glutathione reductase was inactivated. Glutathione oxidation occurred well before cytotoxicity ensued. Addition of ascorbate markedly enhanced SR 4233 cytotoxicity to these compromised hepatocytes.

(2) In contrast, SR 4233 was highly toxic to hypoxic hepatocytes. Addition of ascorbate to enhance SR 4233 reduction also caused a marked increase in hepatocyte toxicity and an SR 4233 radical was detected with ESR spectroscopy. SR 4233 cellular reduction and toxicity was prevented with fructose or inhibitors of NADPH:cytochrome P-450 reductase. Inactivation of catalase or glutathione reductase had no effect on SR 4233 toxicity and hepatocyte GSH was not oxidised indicating oxidative stress did not occur during hypoxic SR 4233 hepatocyte toxicity.

(3) The lack of SR 4233 cytotoxicity under aerobic conditions could probably be attributed to the detoxification of the SR 4233 radical by mitochondrial oxidation as SR 4233, but not its metabolite SR 4317 markedly increased state III and IV mitochondrial respiration in the presence of NADH. The increased respiration was inhibited by the respiratory inhibitors KCN and antimycin A but not by rotenone. Furthermore SR 4233 cytotoxicity under aerobic conditions was markedly increased by partially inhibiting hepatocytes respiration with cyanide but not rotenone.

Over the past two decades there has been a growing effort to develop compounds which are selectively toxic towards radiation resistant hypoxic tumour cells. Hypoxic cell fractions, believed to be present in mammalian solid tumours, are thought to be the reason for the unsuccessful ability to cure some human malignancies by radiotherapy (Moulder & Rockwell, 1987). The benzotriazine di-N-oxide, SR 4233 (3-amino-1,2,4-benzotriazine-1,4-dioxide) is the lead compound of a new series of hypoxic cell cytotoxins currently being investigated as anticancer drugs (Zeman *et al.*, 1989). It is up to 150 times more effective at preventing colony formation if the tumour cells were treated with SR 4233 under hypoxic conditions than under aerobic conditions *in vitro* (Zeman *et al.*, 1986) suggesting that it may be useful in radiation therapy. Recently this has been attributed to chromosome breaks and DNA double strand breaks which are difficult to repair (Wang *et al.*, 1992).

Although the molecular mechanism of toxicity is still being debated it has been proposed that a free radical formed by a one-electron reduction of SR 4233 is the toxic species (Baker *et al.*, 1988; Laderoute *et al.*, 1988) as the two-electron and four-electron reduction metabolites, SR 4317 and SR 4330 did not prevent colony formation (Baker *et al.*, 1988). Toxicity has been postulated to occur via hydrogen abstraction from DNA and other cell constituents by the one-electron reduced SR 4233 free radical (Zeman *et al.*, 1986; Baker *et al.*, 1988) which was presumably detoxified by oxygen. DT-diaphorase isolated from Walker 256 rat tumour cells, was shown to detoxify SR 4233 by catalysing two- and four-electron reduction of SR 4233 (Riley & Workman, 1992).

Recently, White *et al.* (1992) reported that acute dosing of laboratory rats with SR 4233 (0.3 mmol $kg^{-1}$) caused bone marrow toxicity, subcapsular necrosis of the liver as well as necrosis of the kidney medulla and olfactory epithelium. Liver necrosis was confined to hepatocytes of zone 3. The pericentral (Zone 3) area of the liver lobules experience oxygen tensions only in the 2-4% range and hepatocytes at this oxygen tension have been shown to readily metabolise SR 4233 to the stable mono-N-oxide via the radical intermediate (Costa *et al.*, 1989). Furthermore enzymology studies with rat liver microsomes and NADPH have implicated cytochrome P-450 and NADPH:cytochrome P-450 reductase as the major hepatic reductases responsible for the bioactivation of SR 4233 (Walton & Workman, 1990: Walton *et al.*, 1989; Lloyd *et al.*, 1991).

In the following, isolated hepatocytes were chosen as the nonproliferating model target cell using plasma membrane damage as an endpoint. It was found that the resistance of hepatocytes under aerobic conditions to SR 4233 could be attributed to detoxification of the SR 4233 radical by mitochondrial oxidation. Oxidative stress involving GSH oxidation and oxygen activation occurred under aerobic conditions, but was not toxic unless hepatocyte catalase or glutathione reductase was inactivated beforehand. The marked SR 4233 cytotoxicity to hypoxic hepatocytes could be prevented with fructose or NADPH:cytochrome P-450 reductase inhibitors.

### Materials and methods

### *Chemicals*

SR 4233 (3-amino-1,2,4-benzotriazine-1,4-dioxide) was obtained from Dr A.M. Rauth, Ontario Cancer Institute, Toronto, Ontario, Canada and from Dr J.R. Milligan, University of California, CA. SR 4317 (3-amino-1,2,4-benzotriazine-1-N-oxide) and SR 4330 (3-amino-1,2,4-benzotriazine-1) were obtained from Dr M. Tracy, SRI International, Menlo Park, CA. BCNU was a gift from Bristol-Myers Laboratories (Syracuse, N.Y.). Trypan blue, t-butyl hydroperoxide, metyrapone, KCN, GSH, GSSG and sodium azide were obtained from Sigma (St. Louis, MO). Collagenase (from *Clostridium histoliticum*) and HEPES were purchased from Boehringer-Mannheim (Montreal, Quebec). SKF-525A was a gift from Smith Kline Beecham (Oakville, Ontario). Other chemicals were of highest grade available commercially.

Received 28 January 1993; and in revised form 19 April 1993.

*Animals*

Male Sprague-Dawley rats (body wt 220–270 g) fed a standard chow diet and tap water *ad lib* were used to prepare hepatocytes.

*Isolation and incubation of the hepatocytes*

Rat hepatocytes were prepared by collagenase perfusion of the liver as previously described by Moldeus *et al.* (1978). Routinely, 85–95% of the freshly isolated hepatocytes excluded Trypan blue (Trypan blue concentration: 0.2% w/v). The cells were suspended in Krebs-Henseleit buffer containing 12.5 mM HEPES under an atmosphere of 10% $O_2$, 85% $N_2$ and 5% $CO_2$ for 30 min in continuously rotating round bottomed 50 ml flasks at 37°C. The final incubation volume was 10 ml with a concentration of $10^6$ cells $ml^{-1}$. For experiments performed under hypoxic conditions, the cells were incubated under an atmosphere of 95% $N_2$ and 5% $CO_2$ following preincubation (30 min) under aerobic conditions. The oxygen concentration in the cellular medium was <0.1% 30 min after the switch to the hypoxic atmosphere and at this time the experiments were started. SR 4233 and its metabolites were dissolved in dimethyl sulfoxide and added in a final concentration of 0.5% (v/v). The control incubation contained 0.5% (v/v) dimethyl sulfoxide alone.

To inactivate catalase (EC 1.11.1.6) and glutathione reductase (EC 1.6.4.2), sodium azide (final concentration, 4 mM) and BCNU (final concentration: 50 μM) were added respectively to the cells 15 min prior to the start of the experiment (Rossi *et al.*, 1989; Babson & Reed, 1978). Azide and BCNU were not cytotoxic at these concentrations. To oxidise cellular NADPH, t-butyl hydroperoxide (final concentration: 50 μM) was added to hypoxic cells 5 min prior to the start of the experiment (Rush & Alberts, 1986). NADPH levels at this time were decreased by >95% as measured by an HPLC method described by Stocchi *et al.* (1984) and stayed depressed for the duration of the experiment. To inactivate NADPH:cytochrome P-450 reductase, diphenylene iodonium (final concentration: 50μM) or acrolein (final concentration: 100 μM) was added 20 min prior to the start of the experiment (Doussiere & Vignais, 1992; Patel *et al.*, 1984). To inhibit cytochrome P-450, SKF-525A (final concentration: 100 μM) or metyrapone (final concentration: 1 mM) was added to the cells 10 min prior to the start of the experiment (Netter, 1962). To inactivate DT-diaphorase, dicumarol (final concentration: 10 μM) was preincubated for 10 min (Ernster *et al.*, 1960; Rossi *et al.*, 1989). All enzyme modifiers were maintained in the cell medium throughout the experiment and were not cytotoxic at the concentrations used.

*Microsomal and mitochondrial preparation*

Microsomes and mitochondria were isolated from rat liver as previously described (Ernster *et al.*, 1962; Cain & Skilleter, 1987) and kept cold on ice before use. Protein determination was measured by the Bradford method (Bradford, 1976).

*Assays*

Hepatocyte viability was assessed by the Trypan blue dye exclusion test in a Neubauer chamber, by light microscopy. Statistical significance of differences between treated and nontreated groups in these studies were determined by the two-tailed Student *t*-test. The criterion of significance chosen was $P<0.05$.

Total GSH and GSSG in the hepatocyte incubation mixture were measured by HPLC analysis in deproteinated samples (5% metaphosphoric acid) after derivatisation with iodoacetic acid and fluoro-2,4-dinitrobenzene using a μ-Bondapak $NH_2$ column (Waters Model, MA) as described (Reed *et al.*, 1980). GSH and GSSG were used as external standards. A Waters Model 6000A solvent delivery system, equipped with a Waters Model 660 solvent programmer, a WISP 710A automatic injector, and a data module, was used for analysis.

Oxygen consumption was measured by a Clark-type electrode (Model 5300; Yellow-Spring Instrument Co., Inc.) in a 2 ml incubation chamber maintained at 37°C. Prior to oxygen consumption measurements, hepatocytes ($10^6$ cells $ml^{-1}$) were kept at 37°C in Krebs Henseleit buffer, plus HEPES (12.5 mM), pH 7.4 under s stream of 10% $O_2$, 85% $N_2$ and 5% $CO_2$. KCN (2 mM, neutralised with HCl) was added to inhibit mitochondrial respiration. Measurement of isolated mitochondrial respiration was performed with rat liver mitochondria (1 mg $ml^{-1}$) suspended in a respiration buffer containing 0.25 mM sucrose, 5 mM $KH_2PO_4$, 10 mM KCl, 5 mM $MgCl_2$ and 10 mM Tris-HCl at pH 7.4. The respiratory control ratio (RCR) defined as the ratio of oxygen consumption in the presence (state 3) and absence (state 4) of ADP (200 μM), when the substrate concentration is not limiting, was determined by dividing state 3 over state 4 respiration.

Samples for ESR measurements were prepared by the addition of ascorbate (10 mM) to SR 4233 (2 mM) in 0.1 M borax buffer, pH 10, previously bubbled with nitrogen for 10 min. The sample was aspirated into a flat cell and ESR spectra recorded (Mason, 1984). The spectra was scanned several times (usually 8 min scans). The ESR measurements were performed in an aqueous flat cell (8 mm wide) using a TE cavity at room temperature with a Varian E-6 ESR spectrophotometer.

The reduction of SR 4233, and the formation of SR 4317 and SR 4330 in hepatocytes was carried out by HPLC analysis as described by Walton and Workman (Walton & Workman, 1988). Briefly, aliquots (200 μl) of cell samples from the incubation medium ($10^6$ cells $ml^{-1}$) were treated with 2 vol of ice cold methanol and centrifuged at 3000 *g* for 10 min. Supernatant samples were removed and kept at 4°C until injected (100 μl) into the HPLC system for analysis (samples were analysed within 2 h). The isocratic reverse phase chromatography system used comprised of a Beckman 110B pump, a UV detector ERC-7210, a Pharmacia chart recorder, a μ-Bondapak phenyl Rad-Pak column (10 cm × 8 mm, 10-μm beads) under pressure from a Z-module which was protected by a Resolve Cyanopropyl (CN) Guard-Pak precolumn. The mobile phase used was 33% methanol/67% deionised water at a flow rate of 2.5 ml $min^{-1}$. SR 4233, SR 4330 and SR 4317 eluted at 3.4, 5.8 and 7 min, respectively.

### Results

As shown in Table I, SR 4233 (150 μM) added to hepatocytes under 95% $N_2$/5% $CO_2$ caused 100% cell death within a 2 h incubation period as determined by the Trypan blue exclusion test. The viability of untreated hepatocytes incubated under these conditions was not affected. However, under an aerobic environment (10% $O_2$, 85% $N_2$ and 5% $CO_2$), SR 4233 (concentration as high as 2 mM) did not affect hepatocyte viability during the 3 h incubation period.

SR 4233 was cytotoxic under an aerobic environment only when the cell's defence system against oxidative stress was compromised by inactivating hepatocyte catalase with azide or glutathione reductase with BCNU beforehand. As shown in Table I, 1 mM SR 4233 incubated with catalase inactivated or glutathione reductase inactivated hepatocytes caused 78 and 100% cell death, respectively, after a 3 h incubation. Addition of ascorbate to reduce SR 4233 extracellularly markedly increased SR 4233 cytotoxicity towards catalase-inactivated hepatocytes by at least 10-fold (Table I). SR 4233 and ascorbate were not however cytotoxic to normal hepatocytes under aerobic condition. In contrast, under hypoxic conditions, ascorbate increased the susceptibility of normal hepatocytes to SR 4233 and inactivation of catalase or glutathione reductase did not affect hepatocyte susceptibility. Ascorbate by itself was not cytotoxic under hypoxic conditions. These results confirm the selective toxicity of SR 4233 for hypoxic cells and suggests that the cytotoxic

**Table I** SR 4233-induced hepatocyte cytotoxicity under aerobic and hypoxic environments

| *Treatment* | *Cytotoxicity (% Trypan blue uptake)* *Min* 60 | 120 | 180 |
|---|---|---|---|
| *Aerobic conditions* | | | |
| None | 13 ± 2 | 17 ± 3 | 18 ± 3 |
| SR 4233 (2 mM) | 13 ± 2 | 19 ± 3 | 21 ± 3 |
| SR 4233 (1 mM) + azide | 25 ± 4 | 38 ± 4 | 78 ± 5[a] |
| Azide | 13 ± 2 | 18 ± 3 | 21 ± 3 |
| SR 4233 (1 mM) + BCNU | 36 ± 4 | 78 ± 5 | 100[a] |
| BCNU | 14 ± 2 | 18 ± 3 | 22 ± 3 |
| SR 4233 (150 μM) + azide + ascorbate | 41 ± 4 | 85 ± 6 | 100 |
| SR 4233 (150 μM) + KCN | 46 ± 3 | 60 ± 5 | 73 ± 7 |
| KCN | 17 ± 2 | 26 ± 3 | 36 ± 3 |
| *Hypoxic conditions* | | | |
| None | 15 ± 2 | 21 ± 3 | 23 ± 3 |
| SR 4233 (150 μM) | 37 ± 2 | 100[a] | |
| SR 4233 (100 μM) | 24 ± 3 | 36 ± 4 | 55 ± 4[b] |
| SR 4233 (100 μM) + ascorbate | 56 ± 4 | 100[a] | |
| SR 4233 (100 μM) + azide | 25 ± 4 | 39 ± 4 | 59 ± 5[b] |
| SR 4233 (100 μM) + BCNU | 24 ± 4 | 40 ± 4 | 61 ± 5[b] |
| SR 4233 (100 μM) + DPI | 18 ± 2 | 22 ± 2 | 28 ± 3 |
| SR 4233 (150 μM) + fructose | 16 ± 2 | 21 ± 3 | 25 ± 3 |
| SR 4233 (150 μM) + fructose + monensin | 20 ± 3 | 45 ± 4 | 86 ± 5[a] |
| SR 4233 (150 μM) + monensin | 39 ± 3 | 100 | |
| Monensin | 15 ± 2 | 22 ± 3 | 22 ± 3[a] |

Hepatocytes ($10^6$ cells $ml^{-1}$) were preincubated in Krebs-Henseleit buffer, pH 7.4 at 37°C. Cells were maintained under an aerobic or hypoxic environment as described in Materials and methods. SR 4233 was then added to the incubation mixture. Where stated azide (4 mM), monensin (10 μM), DPI (50 μM), fructose (20 mM) and KCN (400 μM) were preincubated for 5 min prior to SR 4233 addition. BCNU (50 μM) however, was preincubated for 20 min prior to SR 4233 addition. Cell toxicity was determined as the percentage of cells taken up Trypan blue. [a]Significantly different from untreated cells ($P < 0.001$). [b]Significantly different from untreated cells ($P < 0.02$).

Figure 1 GSH depletion **a**, and GSSG formation **b**, induced by SR 4233 in isolated hepatocytes under aerobic conditions. Hepatocytes ($10^6$ cells $ml^{-1}$) were incubated alone or with 2 mM SR 4233 (○), with azide (4 mM) + SR 4233 (1 mM) (□), with BCNU (50 μM) + SR 4233 (●) and with ascorbate (10 mM) + azide (4 mM) + SR 4233 (0.5 mM) (■). GSH and GSSG levels were determined by HPLC analysis, as described in 'Materials and methods'. Three separate experiments were carried out. Points, mean; bars, s.e.

mechanisms for toxicity under aerobic *vs* hypoxic environments are different.

### *Effect of SR 4233 on hepatocyte glutathione*

Additon of SR 4233 to isolated hepatocytes under either aerobic or hypoxic conditions did not affect the levels of intracellular GSH (Figure 1). However, intracellular GSH was oxidised when SR 4233 was added to catalase or glutathione reductase inactivated hepatocytes under aerobic conditions. (Figure 1). Intracellular GSH levels in these compromised hepatocytes were little affected over the time period studied in the absence of SR 4233 (results not shown). Glutathione reductase-inactivated cells had lower initial intracellular GSH levels due to conjugate formation by a metabolite of BCNU. Furthermore, the addition of ascorbate to catalase deficient cells markedly enhanced GSH oxidation induced by SR 4233 and a 4-fold lower SR 4233 concentration was effective (Figure 1). Ascorbate by itself had no effect on GSH levels in catalase deficient cells under aerobic conditions. Under hypoxic conditions GSH levels in catalase-deficient or glutathione reductase inactivated cells were also not affected by ascorbate (results not shown).

*SR 4233-dependent oxygen consumption by isolated hepatocytes and by rat liver microsomes and mitochondria*

Even though SR 4233 was not cytotoxic to hepatocytes under aerobic conditions, cyanide-resistant respiration was induced in hepatocytes on addition of SR 4233. In order to investigate the ability of SR 4233 to undergo one-electron reduction and redox cycle with molecular oxygen, ascorbate was used to stimulate reduction. As shown in Table II, addition of ascorbate to reduce SR 4233 was accompanied by marked oxygen consumption. $H_2O_2$ was formed as addition of catalase at the end of the experiment caused an immediate release of oxygen (results not shown).

ESR studies showed that an SR 4233 radical was formed upon incubation of SR 4233 (2 mM) with ascorbate (10 mM) at pH 10 under an atmosphere of $N_2$ (Figure 2a). In the absence of SR 4233 or ascorbate, the radical was not formed (Figure 2b and c). When the sample in the flat cell was de-aspirated and exposed to air, the SR 4233 radical immediately disappeared and was detected again after rebubbling the sample with nitrogen. Neither SR 4317 or SR 4330 produced detectable radicals nor stimulated oxygen consumption when added to ascorbate (results not shown).

Addition of SR 4233 to rat liver microsomes in the presence of NADPH also stimulated oxygen consumption. Interestingly, incubation of SR 4233 with respiring rat liver mitochondria induced NADH-dependent state 3 and 4 mitochondrial respiration which was completely inhibited by KCN and antimycin A but not by rotenone (Table III). SR 4233 or NADH alone did not induce mitochondrial respiration. Also, incubation of mitochondria with either SR 4317 or SR 4330 did not induce any NADH dependent mitochondrial respiration. This indicated that metabolites of SR 4233 can act as substrates for the mitochondiral respiratory chain. Furthermore, this suggests that mitochondria may play a protective role against SR 4233-induced toxicity in the intact cell by detoxifying the free radical via the mitochondrial respiratory chain. In support of this theory SR 4233 became nearly as cytotoxic to hepatocytes under aerobic conditions as hepatocytes under hypoxic conditions if a nontoxic concentration of KCN (400 μM) was added to partly inhibit mitochondrial electron transport (Table I). At this KCN concentration hepatocyte respiration was inhibited 76–78% (results not shown). Furthermore SR 4233 was not cytotoxic to hepatocytes treated with rotenone (5 μM) to inhibit mitochondrial respiration as shown in Table III (results not shown).

*Metabolism of SR 4233 by isolated hepatocytes*

HPLC analysis was used to determine the reduction of SR 4233 by isolated hepatocytes under hypoxic conditions. As shown in Table IV, SR 4233 (150 μM) was rapidly metabolised within 1 h at a rate of 3.4 ± 0.6 mmol $min^{-1}$ $10^6$ cells to form SR 4317. No SR 4330 was formed during this incubation period. In aerobic hepatocyte incubations no loss of SR 4233 occurred during a 1 h incubation period (results not shown). Addition of SKF-525A or metyrapone, two well known inhibitors of cytochrome P-450, or the DT-diaphorase inhibitor, dicumarol (Ernster *et al.*, 1960), had no effect on the rate of SR 4233 metabolism by hypoxic hepatocytes suggesting that these two enzymes do not play a major role in the reduction of SR 4233 by hepatocytes.

NADPH:cytochrome P-450 reductase has been suggested to be involved in the one-electron reduction of SR 4233 to a free radical which in the absence of oxygen may be further reduced to the mono-oxide, SR 4317 (Walton & Workman, 1990; Walton *et al.*, 1989; Lloyd *et al.*, 1991). In order to investigate this hypothesis in our hypoxic cell model we monitored SR 4233 reduction in NADPH oxidised hepatocytes. To oxidise cellular NADPH, a small nontoxic dose (50 μM) of t-butyl hydroperoxide (t-BHP) was added to the cell incubation mixture prior to SR 4233 addition (Rush & Alberts, 1986). NADPH levels after this treatment were depressed by >95% for the duration of the experiment. As shown in Table IV, the metabolism of SR 4233 by hypoxic hepatocytes was completely inhibited by the presence of t-BHP.

As yet no inhibitors of NADPH:cytochrome P-450 reductase have been successfully used to inactivate the reductase in intact cells without causing toxicity. However, diphenylene iodonium which has been recently reported to inactivate

**Table II** SR 4233-induced oxygen uptake by ascorbate, microsomes, mitochondria and isolated hepatocytes

| *Addition (cells)* | *Ascorbate (nmol min⁻¹)* | *Oxygen consumed — Microsomes NADPH (nmol min⁻¹ mg⁻¹)* | *Hepatocytes (nmol min⁻¹ 10⁻⁶) + KCN* |
|---|---|---|---|
| SR 4233 500 μM | 26.4 ± 4.4 | 16.4 ± 3.5 | 9.3 ± 1.3 |
| SR 4233 250 μM | 18.5 ± 2.4 | 10.5 ± 2.2 | 4.5 ± 1.4 |
| SR 4233 100 μM | 7.5 ± 1.3 | n.d.[a] | n.d.[a] |

Oxygen uptake was measured using a Clarke-type electrode as described in Materials and methods. SR 4233 was added to either ascrobate (10 mM) in 0.1 M Tris-HCl buffer pH 7.4 alone or to isolated microsomes (1 mg $ml^{-1}$) + 1 mM NADPH at 37°C. SR 4233-induced oxygen uptake in hepatocytes was monitored in Krebs-Henseleit buffer, containing HEPES (12.5 mM) pH 7.4 at a cell density of $10^6$ cells $ml^{-1}$ and 37°C in the presence of KCN (2 mM). Values are expressed as means of three separate experiments (± s.e.). [a]Not determined.

**Table III** Effect of SR 4233 administration on mitochondrial respiration[a]

| *Treatment* | *State 3* | *State 4* | *RCR*[b] |
|---|---|---|---|
| Succinate | 76.6 ± 5.9 | 20.7 ± 2.2 | 3.7 |
| SR 4233 (250 μM) + NADH (1 mM) | 6.5 ± 1.6 | 3.2 ± 0.5 | 2.0 |
| SR 4233 (500 μM) + NADH (1 mM) | 12.4 ± 2.1 | 5.3 ± 0.8 | 2.3 |
| + KCN (1 mM) | 0 | 0 | |
| + Antimycin A (5 μM) | 0 | 0 | |
| + Rotenone (5 μM) | 11.3 ± 2.2 | 5.5 ± 0.9 | 2.0 |
| SR 4317 (250 μM) + NADH (1 mM) | 0 | 0 | |
| SR 4330 (250 μM) + NADH (1 mM) | 0 | 0 | |

[a]nmol oxygen uptake $min^{-1}$ $mg^{-1}$ protein. [b]RCR, respiratory control ratio. Note: Experiments were performed as described in Materials and methods. Values are expressed as the means of three separate experiments (± s.d.).

**Figure 2** The ESR spectrum of the SR 4233 radical generated in a system (0.1 M borax buffer, pH 10) of (2 mM) and ascorbate (10 mM) previously bubbled with nitrogen for 10 min. (**b**), same as in **a**, but without SR 4233, **c**, same as in **a**, but without ascorbate. The instrumental conditions were: 20-mW microwave power, 12.5 G $min^{-1}$ scan rate, 0.5-s time constant, 1.6 G modulation amplitude, and $5 \times 10^4$ receiver gain.

NADPH:cytochrome P-450 reductase provided the enzyme was preincubated with NADPH (Doussiere & Vignais, 1992), prevented SR 4233 metabolism (Table IV) and cytotoxicity (Table I) in hepatocytes. Acrolein has also been reported to selectively inactivate NADPH:cytochrome P-450 reductase (Patel *et al.*, 1984). Addition of a non cytotoxic concentration of acrolein to the hepatocytes also markedly delayed SR 4233 metabolism (Table IV).

**Table IV** Metabolism of SR 4233 by hypoxic isolated hepatocytes

| *Addition* | *Rate of SR 4233 disappearance (nmol $min^{-1}$ $10^{-6}$ cells)* |
|---|---|
| SR 4233 (150 μM) | 3.4 ± 0.6 |
| SR 4233 (150 μM) + acrolein (100 μM) | 1.4 ± 0.2[a] |
| SR 4233 (150 μM) + t-BHP (50 μM) | 0 |
| SR 4233 (150 μM) + DPI (50 μM) | 0.8 ± 0.2 |
| SR 4233 (150 μM) + fructose (10 mM) | 3.2 ± 0.6 |

Hepatocytes ($10^6$ cells $ml^{-1}$) were incubated in Krebs-Henseleit buffer, containing HEPES (12.5 mM) pH 7.4 at 37°C under hypoxic conditions. Where stated either acrolein fructose, DPI or t-BHP was preincubated for approximately 5 min prior to SR 4233 addition. SR 4233 and the product SR 4233 were measured after 20 min of cell incubation by HPLC analysis as described in Materials and methods. Values are expressed as means of three separate experiments ( ± s.e.). [a]Significantly different from SR 4233 alone ($P < 0.05$).

*Prevention of SR 4233 cytotoxicity towards hypoxic hepatocytes*

As shown in Table I diphenylene iodonium was the most effective NADPH:cytochrome P-450 reductase inhibitor used which prevented SR 4233 cytotoxicity in hypoxic isolated hepatocytes. Fructose also prevented SR 4233 cytotoxicity. However as shown in Table IV, SR 4233 reduction by hepatocytes was not affected. As fructose markedly induces glycolysis and acidosis in hypoxic hepatocytes (Seglen, 1974) or perfused liver (Iles *et al.*, 1980) and as an acidotic pH protects against hypoxic hepatocyte injury (Gores *et al.*, 1989), the protective effect of acidosis against SR 4233 cytotoxicity was investigated. As shown in Table IV monensin, an agent which catalyses the exchange of $Na^+$ for $H^+$ and equalises intracellular pH to that of extracellular pH (Gores *et al.*, 1989), prevented fructose protection. Furthermore extracellular acidosis (pH 6.5) prevented SR 4233 cytotoxicity towards hypoxic hepatocytes without affecting SR 4233 metabolism (results not shown).

### Discussion

In this paper we confirm that the mechanism of SR 4233-induced cytotoxicity differs in aerobic *vs* hypoxic environments. SR 4233 is shown to be metabolised in isolated hepatocytes by NADPH:cytochrome P-450 reductase resulting in cellular toxicity in the absence of oxygen which can probably be attributed to the SR 4233 radical. However in the presence of oxygen, SR 4233 was shown to redox cycle resulting in nontoxic oxidative stress. SR 4233 was also shown to redox cycle via the mitochondrial electron transport system suggesting a new mechanism for detoxification of the SR 4233 radical under aerobic condition without oxygen activation resulting in oxidative stress.

SR 4233 has been suggested to undergo a one electron reduction by various enzymes including NADPH:cytochrome P-450 reductase. This generates a free radical which either undergoes a futile redox cycle with molecular oxygen or under hypoxia may disproportionate and/or acquire a second electron via hydrogen abstraction to form a stable nontoxic mono-oxide, SR 4317 (Laderoute *et al.*, 1988; Walton & Workman, 1990; Walton *et al.*, 1989; Lloyd *et al.*, 1991). In the present work ascorbate was also found to be very efficient at inducing oxygen consumption in the presence of SR 4233 but not the SR 4233 reduction metabolites, SR 4317 or SR4330. This suggests that SR 4233 is reduced to a free radical by ascorbate which in the presence of oxygen redox cycles back to the parent compound hence consuming oxygen in the process. Addition of catalase at the end of the experiment caused a recovery in oxygen indicating that $O_2^-$ and $H_2O_2$ had been formed, presumably from the ascorbate catalysed SR 4233 reduction. ESR evidence also showed the production of a free radical signal when ascorbate reduced SR 4233. Even though we were not able to identify the free radical obtained from the ESR spectra, a recent study by Lloyd *et al.* (1991) identified a nitroxyl radical formed by incubation of a liver microsomal system containing NADPH with SR 4233. Moreover, using DMPO spin trap experiments they also showed that superoxide radicals are formed in the reaction.

The induction of cyanide-resistant respiration when SR 4233 was added to hepatocytes suggests that hepatocytes are also able to catalyse the reduction of SR 4233 to a free radical which in turn redox cycles in the presence of oxygen thereby exposing the cells to oxidative stress. Mammalian cells are usually well equipped with a defence mechanism system against oxidative stress. This system includes enzymes such as superoxide dismutase, glutathione peroxidase, glutathione reductase, catalase and reducing agents such as GSH. Previously, we have shown in isolated rat hepatocytes that the toxic potency of compounds, such as nitrofurantoin and diaziquone which undergo intracellular futile redox-cycling with oxygen to generate $H_2O_2$, was markedly increased if components of the cell's peroxide defence system, such as catalase, were inhibited (Rossi *et al.*, 1989; Silva & O'Brien, 1989). Furthermore, $H_2O_2$ added directly to the incubation medium was toxic only to catalase-inactivated cells (Rossi *et al.*, 1989). As shown in this paper, SR 4233 toxicity to hepatocytes under aerobic conditions was observed only when the cell's defence system against oxidative stress was compromised by either inhibition of catalase or glutathione reductase. Addition of ascorbate to catalase-inactivated hepatocytes further increased the susceptibility of these cells to SR 4233-induced cytotoxicity. Under these conditions intracellular GSH was rapidly oxidised to GSSG well before cell death occurred. These results indicate that SR 4233 causes oxidative stress under aerobic conditions. Moreover, HPLC analysis of SR 4233 following incubation with hepatocyte under aerobic conditions did not show any loss of the parent compound. This provides further evidence that SR 4233 undergoes futile redox-cycling in hepatocytes under an aerobic environment.

Under hypoxic conditions, SR 4233 was at least 50-fold more toxic to hepatocytes compared to aerobic conditions. Toxicity occurred without affecting the cell's GSH levels. Addition of ascorbate to enhance the reduction rate of SR 4233 increased its toxic potency. In contrast to the aerobic study, inactivation of either catalase or glutathione reductase under hypoxic conditions did not affect SR 4233 induced toxicity or intracellular GSH levels. These results suggest that the mechanism of SR 4233-induced hepatocyte toxicity differs in aerobic *vs* hypoxic environments.

HPLC analysis confirmed that SR 4233 is rapidly metabolised by hypoxic hepatocytes within 1 h of incubation with the formation of the stable two-electron reduction metabolite, SR 4317, being the only metabolite observed. SR 4317 added to the hypoxic cells, did not undergo further reduction or cause any loss in cell viability. SR 4233 metabolism was prevented if cellular NADPH was oxidised with a pulse of t-butyl hydroperoxide (t-BHP). The latter oxidises hepatocyte NADPH because glutathione peroxidase and glutathione reductase are involved in the reductive metabolism of t-BHP (Rush & Alberts, 1986). Pretreatment of the cells with diphenylene iodonium, an NADPH:cytochrome P-450 reductase inhibitor (Doussiere & Vignais, 1992), prevented SR 4233 reduction and cytoxicity. In a previous study with anaerobic mouse liver microsomes, cytochrome P-450 was implicated in the reduction of SR 4233 because carbon monoxide inhibited the liver microsomal (phenobarbital induced) catalysed metabolism of SR 4233 by 78–86% (Walton & Workman, 1990). DT-diaphorase was also found to reduce SR 4233 (Riley & Workman, 1992). However, in our study the inhibition of hepatocyte cytochrome P-450 by SKF-525A or metyrapone or the inactivation of DT-diaphorase by dicumarol did not affect SR 4233 reduction. Furthermore, Lloyd *et al.* (1991) showed that pretreating rat liver microsomes with metyrapone or carbon monoxide had no effect on the production of SR 4233 radicals. This also suggests that NADPH:cytochrome P-450 reductase is the major reductase in the metabolism of SR 4233 by the uninduced liver. However our results do not rule out the involvement of cytochrome P-450 in SR 4233 reduction in hepatocytes isolated from rats after the administration of various cytochrome P-450 inducers.

Incubation of hepatocytes under hypoxic conditions with fructose inhibited SR 4233-induced cell death without affecting its reduction. Other investigators have shown that fructose markedly increased the anaerobic production of lactic acid particularly in hypoxic hepatocytes (Seglen, 1974), the classical Pasteur effect, and that significant acidosis developed (Iles *et al.*, 1980). Furthermore acidosis protects against hypoxic injury in hepatocytes (Gores *et al.*, 1989). Because of this the effect of monensin, an agent which catalyses the exchange of $Na^+$ for $H^+$ and has been shown to equalise intracellular pH to that of extracellular pH (Gores *et al.*, 1989) on the fructose protection was investigated. It was found that monesin prevented the antidotal effect of fructose. As perfusion of the liver with fructose also causes acidification of the medium (Sies & Noack, 1972) the effect of decreasing the pH of the medium on SR 4233 hypoxic cytotoxicity was investigated. SR 4233 was found to be much less cytotoxic to hypoxic hepatocytes at pH 6.5 than at pH 7.4 (results not shown). This suggests that the protective effect of fructose under hypoxic conditions can be attributed to acidosis.

Recently Riley and Workman (1992) reported that DT-diaphorase purified from Walker 256 rat tumour cells catalyses a direct two- and four-electron reduction of SR 4233. However, our studies indicate that dicumarol, a highly effective inhibitor of DT-diaphorase had no effect on SR 4233 reduction by hypoxic hepatocytes. SR 4233 radical formation by NADPH and rat liver microsomes has also recently been reported to be unaffected by dicumarol (Lloyd *et al.*, 1991). Furthermore, the NADPH:cytochrome P-450 reductase inhibitor, diphenylene iodonium, did not inhibit DT-diaphorase (results not shown) but SR 4233 reductive metabolism was inhibited and cytotoxicity was prevented.

Previous studies with hypoxic primary cultured hepatocytes and CHO cells have correlated the reduction of SR 4233 with its toxic potency. The reduction of SR 4233

and its toxic potency were dramatically decreased under aerobic conditions (Baker *et al.*, 1988; Riley & Workman, 1992). SR 4233 also induced cyanide-resistant respiration in CHO cells (Baker *et al.*, 1988). However, they did not address the mechanism responsible for the aerobic toxicity of SR 4233. Our studies with isolated hepatocytes support the proposal that the SR 4233 free radical is formed under both aerobic and hypoxic conditions but suggests that under aerobic conditions SR 4233 would be cytotoxic to peroxide susceptible cells.

The primary reason suggested for the high selectivity of SR 4233 toxicity against hypoxic cells *vs* aerobic cells is the back oxidation of the SR 4233 free radical by molecular oxygen. In the absence of oxygen the free radical is readily available to interact with the cell's macromolecules resulting in toxicity. However, in our present study, an additional mechanism was discovered involving detoxification of the radical as result of oxidation by the mitochondrial electron transport system. Using freshly isolated rat liver mitochondria, SR 4233 was also shown to support mitochondrial respiration in the presence of NADH. Mitochondrial respiration was completely inhibited by KCN and antimycin A but not by rotenone. The lack of inhibition by rotenone suggest that the site of interaction involves ubiquinone and the cytochromes. The reductive metabolites SR 4317 and SR 4330, did not stimulate mitochondrial respiration. The requirement of NADH suggests that the SR 4233 radical formed by mitochondrial outer membrane reductases can act as an electron donor for the mitochondrial electron transport chain. Many semiquinone radicals are also known to rapidly reduce cytochrome C (O'Brien, 1991) and can therefore be detoxified by mitochondrial oxidation. Oxidation of the free radical by ubiquinone or cytochromes under aerobic conditions would also prevent interaction of the radical with critical sites in the cell (Winterbourn, 1981). Under hypoxia, any free radicals produced from the incubation of hepatocytes with SR 4233 would no longer be detoxified by oxidation by the mitochondrial electron transport system as ubiquinone and cytochromes are now fully reduced. In support of this theory, hepatocytes treated with cyanide to inhibit mitochondrial respiration by 76–78% were nearly as susceptible to SR 4233 under aerobic conditions as hypoxic hepatocytes. However it is also possible that partly depleting hepatocyte ATP levels with cyanide also increases SR 4233 toxicity. Although SR 4233 did not affect ATP levels in aerobic or hypoxic cells (results not shown). Furthermore the low ATP levels in hypoxic hepatocyte may also contribute to their marked susceptibility to SR 4233.

In conclusion the present study using isolated hepatocytes confirms previous work with other cell systems in establishing SR 4233 as a selective hypoxic cell cytotoxin and also provides evidence for the first time of an oxygen dependent SR 4233-mediated oxidative stress which is nontoxic in the cells with peroxide metabolising enzymes. At the same time it is shown that both oxygen and mitochondria may prevent SR 4233 mediated toxicity by reoxidising the free radical metabolite of SR 4233.

**Abbreviations:** GSH, glutathione; GSSG, oxidised glutathione; HEPES, 4-(2-hydroxyethyl)-1-piperazineethane-sulfonic acid; ESR, electron spin resonance; DMSO, dimethyldisulfoxide; BCNU, N,N-bis(2-chloroethyl)-N-nitrosourea; KCN, potassium cyanide; s.e., standard error.

### References

BABSON, J. & REED, D. (1978). Inactivation of glutathione reductase by 2-chloroethylnitrosourea-derived isocyanates. *Biochem. Res. Commun.*, **83**, 754–762.

BAKER, M.A., ZEMAN, E.M. HIRST, V.K. & BROWN, J.M. (1988). Metabolism of SR 4233 by Chinese hamster ovary cells: basis of selective hypoxic cytotoxicity. *Cancer Res.*, **48**, 5947–5952.

BRADFORD, M.M. (1976). A rapid and sensitive method for the quantification of microgram quantities of protein utilizing the principle of protein dye binding. *Anal. Biochem.*, **72**, 248–256.

CAIN, K. & SKILLETER, D.N. (1987). Preparation and use of mitochondria in toxicological research. In *Biochemical Toxicology: A Practical Approach.*, Snell, K. & Mullock, B. (eds). Washington: IRL Press, pp. 217–223.

COSTA, A.K., BAKER, M.A., BROWN, J.M. & TRUDELL, J.R. (1989). *In vitro* hepatotoxicity of SR 4233 (3-amino-1,2,4-benzotriazine-1,4-dioxide), a hypoxic cytotoxin and potential antitumor agent. *Cancer Res.*, **49**, 925–929.

DOUSSIERE, J. & VIGNAIS, P.V. (1992). Diphenylene iodonium as an inhibitor of the NADPH oxidase complex. *Eur. J. Biochem.*, **208**, 61–71.

ERNSTER, L., LJUNGGREN, M. & DANIELSON, L. (1960). Purification and some properties of a highly dicumarol-sensitive liver diaphorase. *Biochem. Biophys. Res. Comm.*, **2**, 88–92.

ERNSTER, L., SUKEVITZ, P. & PALADE, G.E. (1962). Enzyme relationships in endoplasmic reticulum of rat liver. *J. Cell Biol.*, **15**, 541–562.

GORES, G.J., NIEMINEN, A., WRAY, B., HERMAN, B. & LEMASTERS, J. (1989). Intracellular pH during 'chemical hypoxia' in cultured rat hepatocytes. Protection by intracellular acidosis against the onset of cell death. *J. Clin. Invest.*, **83**, 386–396.

ILES, R.A., GRIFFITHS, J.R., STEVENS, A., GADIAN, D.G. & PORTEOUS, R. (1980). Effects of fructose on the energy metabolism and acid-base status of the perfused starved rat-liver. *Biochem. J.*, **192**, 191–202.

LADEROUTE, K., WARDMAN, P. & RAUTH, A.M. (1988). Molecular mechanisms for the hypoxic-dependent activation of 3-amino-1,2,4-benzotriazine-1,4-dioxide (SR 4233). *Biochem. Pharmacol.*, **37**, 1487–1495.

LLOYD, R.V., DULING, D.R., RUMYANTSEVA, G.V., MASON, R.P. & BRIDSON, P.K. (1991). Microsomal reduction of 3-amino-1,2,4-benzotriazene 1,4-dioxide to a free radical. *Mol. Pharmacol.*, **40**, 440–445.

MASON, R.P. (1984). Assay of *in situ* radicals by electron spin resonance. *Methods Enzymol.*, **105**, 416–422.

MOLDEUS, P., HOGBERG, H. & ORRENIUS, S. (1978). Isolation and use of liver cells. *Methods Enzymol.*, **52**, 60–71.

MOULDER, J. & ROCKWELL, S. (1987). Tumour hypoxia: its impact on cancer therapy. In *Cancer Metastasis Reviews*, Vol. 5, Fidler, I.J. & Poste, G. (eds). Boston: Nijhoff Publishing, pp. 313–341.

NETTER, K.J. (1962). Drugs as inhibitors of drug metabolism. In *Proceedings of the First International Pharmacology Meeting*, vol. 6, B. Unvas (ed.) New York: Macmillan Co., pp. 213–228.

O'BRIEN, P.J. (1991). Molecular mechanisms of quinone cytotoxicity. *Chem. Biol. Interact.*, **80**, 1–41.

PATEL, J.M., ORTIZ, E., KOLMSTETTER & LEIBMAN, K.C. (1984). Selective inactivation of rat lung and liver microsomal NADPH-cytochrome c reductase by acrolein. *Drug Metab. Dispos.*, **12**, 460–463.

REED, D.J., BABSON, J.R., BRODIE, A.E., ELLIS, W.W. & POTTER, D.W. (1980). High performance liquid chromatography analysis of nanomole levels of glutathione, glutathione disulfide and related thiols and disulfides. *Anal. Biochem.*, **106**, 55–62.

RILEY, R.J. & WORKMAN, P. (1992). Enzymology of the reduction of the benzotriazine-di-N-oxide hypoxic cell cytotoxin SR 4233 (WIN 59075) by NAD(P)H::(quinone acceptor) oxidoreductase (EC 1.6.99.2) purified from Walker 256 rat tumour cells. *Biochem. Pharmacol.*, **43**, 167–174.

ROSSI, L., SILVA, J.M., MCGIRR, L.G. & O'BRIEN, P.J. (1989). Nitrofurantoin-mediated oxidative stress in isolated rat hepatocytes. *Biochem. Pharmacol.*, **37**, 3109–3117.

RUSH, G.F. & ALBERTS, D. (1986). tert-Butyl hydroperoxide metabolism and stimulation of the pentose phosphate pathway in isolated rat hepatocytes. *Toxicol. Appl. Pharmacol.*, **85**, 324–331.

SEGLEN, P.O. (1974). Autoregulation of glycolysis, respiration, gluconeogenesis and glycogen synthesis in isolated parenchymal rat liver cells under aerobic and anaerobic conditions. *Biochim. Biophys. Acta*, **338**, 317–336.

SIES, H. & NOACK, G. (1972). Proton movement accompanying monocarboxylate permeation in hemoglobin free perfused rat liver. *FEBS Lett.*, **22**, 193–196.

SILVA, J.M. & O'BRIEN, P.J. (1989). Diaziquone-induced cytotoxicity in isolated rat hepatocytes. *Cancer Res.*, **49**, 5550–5554.

STOCCHI, V., CUCCHIARINI, L., MAGNANI, M., CHIARANTINI, L., PALMA, P. & CRESCENTINI, G. (1984). Simultaneous extraction and reverse-phase high performance liquid chromatography determination of adenine and pyridine nucleotides in human red blood cells. *Anal. Biochem.*, **146**, 118-124.

WALTON, M.I. & WORKMAN, P. (1988). High-performance liquid chromatography assay for the benzotriazine di-N-oxide (SR 4233) and its reduced metabolites in biological materials. *J. Chromatogr.*, **430**, 4429-4437.

WALTON, M.I., WOLF, C.R. & WORKMAN, P. (1989). Molecular enzymology of the reductive bioactivation of hypoxic cell cytotoxins. *Int. J. Radiat. Oncol. Biol. Phys.*, **16**, 983-986.

WALTON, M.I. & WORKMAN, P. (1990). Enzymology of the reductive bioactivation of SR 4233. A novel benzotriazine di-N-oxide hypoxic cell cytotoxin. *Biochem. Pharmacol.*, **39**, 1735-1742.

WANG, J., BIEDERMANN, K.A. & BROWN, J.M. (1992). Repair of DNA and chromosome breaks in cells exposed to SR 4233 under hypoxia or to ionising radiation. *Cancer Res.*, **52**, 4473-4477.

WHITE, I.N., CAHILL, A., DAVIS, A. & CARTHEW, P. (1992). Acute lesions in rats caused by 3-amino-1,2,4-benzotriazine-1,4-dioxide (SR 4233) or nitromin: a comparison with rates of reduction in microsomal systems from target organs. *Arch. Toxicol.*, **66**, 100-106.

WINTERBOURN, C.C. (1981). Cytochrome C reduction by semiquinone radicals can be directly inhibited by superoxide dismutase. *Arch. Biochem. Biophys.*, **209**, 159-167.

ZEMAN, E.M., BROWN, J.M., LEMMON, M.J., HIRST, B.K. & LEE, W.W. (1986). SR 4233: a new bioreductive agent with high selective toxicity for hypoxic mammalian cells. *Int. J. Radiat. Oncol. Biol. Phys.*, **12**, 1239-1942.

ZEMAN, E.M., BAKER, M.A., LEMMON, M.J., PEARSON, C.I., ADAMS, J.A., BROWN, J.M., LEE, W.W. & TRACY, M. (1989). Structure-activity relationships for benzotriazine di-N-oxides. *Int. J. Radiat. Biol. Phys.*, **16**, 977-981.